# *N* ^2^-[1-(2-Hydroxy­phen­yl)ethyl­idene]-*N* ^2′^-(1*H*-indol-3-ylmethyl­ene)carbonic dihydrazide

**DOI:** 10.1107/S1600536808035757

**Published:** 2008-11-13

**Authors:** Siti Munirah Saharin, Hapipah Mohd Ali, Ward T. Robinson, A. A. Mahmood

**Affiliations:** a Department of Chemistry, University of Malaya, 50603 Kuala Lumpur, Malaysia; b Department of Molecular Medicine, Faculty of Medicine, University of Malaya, 50603 Kuala Lumpur, Malaysia

## Abstract

In the crystal structure of the title compound {alternative name: 1-[1-(2-hydroxy­phen­yl)ethyl­ideneamino]-3-(1*H*-indol-3-ylmethyl­eneamino)urea}, C_18_H_17_N_5_O_2_, the planar indole component is twisted at an angle of 63.7 (10)° with respect to the rest of the mol­ecule. This compound is one of a series being studied for biological activity. The hydr­oxy groups are involved in both intra­molecular (O—H⋯N) and inter­molecular (N—H⋯O) hydrogen bonds.

## Related literature

For a related compound, see: Dan *et al.* (1987 ▶).
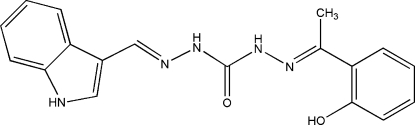


## Experimental

### 

#### Crystal data

C_18_H_17_N_5_O_2_
*M* *_r_* = 335.37
Monoclinic, 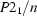

*a* = 7.0802 (8) Å
*b* = 9.5335 (11) Å
*c* = 25.110 (3) Å
β = 97.295 (2)°
*V* = 1681.2 (3) Å^3^
*Z* = 4
Mo *K*α radiation
μ = 0.09 mm^−1^
*T* = 100 (2) K
0.42 × 0.42 × 0.16 mm

#### Data collection

Bruker APEXII CCD area-detector diffractometer
Absorption correction: multi-scan (*SADABS*; Sheldrick, 1996 ▶) *T*_min_ = 0.821, *T*_max_ = 0.986
11912 measured reflections
4740 independent reflections
4145 reflections with *I* > 2σ(*I*)
*R*_int_ = 0.014

#### Refinement

*R*[*F* ^2^ > 2σ(*F* ^2^)] = 0.043
*wR*(*F* ^2^) = 0.122
*S* = 1.03
4740 reflections
228 parameters
H-atom parameters constrained
Δρ_max_ = 0.43 e Å^−3^
Δρ_min_ = −0.32 e Å^−3^

### 

Data collection: *APEX2* (Bruker, 2007 ▶); cell refinement: *SAINT* (Bruker, 2007 ▶); data reduction: *SAINT*; program(s) used to solve structure: *SHELXS97* (Sheldrick, 2008 ▶); program(s) used to refine structure: *SHELXL97* (Sheldrick, 2008 ▶); molecular graphics: *X-SEED* (Barbour, 2001 ▶); software used to prepare material for publication: *publCIF* (Westrip, 2008 ▶).

## Supplementary Material

Crystal structure: contains datablocks I, global. DOI: 10.1107/S1600536808035757/hg2434sup1.cif

Structure factors: contains datablocks I. DOI: 10.1107/S1600536808035757/hg2434Isup2.hkl

Additional supplementary materials:  crystallographic information; 3D view; checkCIF report

## Figures and Tables

**Table 1:** Hydrogen-bond geometry (Å, °)

*D*—H⋯*A*	*D*—H	H⋯*A*	*D*⋯*A*	*D*—H⋯*A*
O1—H1*A*⋯N5	0.84	1.78	2.5121 (11)	145
N3—H3*B*⋯O2^i^	0.88	1.99	2.8481 (13)	166
N1—H1*B*⋯O1^ii^	0.88	2.14	2.8803 (13)	142

Symmetry codes: (i) 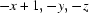; (ii) 
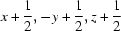
.

**Fig. 1.**
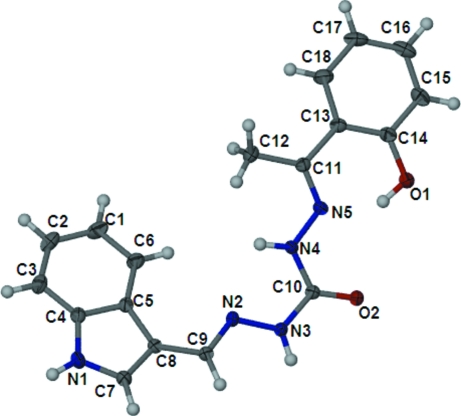
Thermal ellipsoid plot (Barbour, 2001) of C18H17N5O2 at 70% probability level. Hydrogen atoms are drawn as spheres of arbitrary radius

## supplementary crystallographic information

### Comment

X-ray structures, of Schiff bases derived from condensation of indole-3-carboxaldehyde and 2-hydroxyacetophenone, have not been investigated. The title compound (Fig. 1) appears to be the first example with the planar indole component is twisted at an angle of 116.3 (10)° with respect to the rest of the molecule. However, compound bis(salicylidene)carbonohydrazide (Dan *et al.*1987), which was reported previously shows planarity for the whole molecule.

### Experimental

Indole-3-carboxaldehyde (0.30 g, 2.07 mmol), carbohydrazide (0.187 g, 2.07 mmol), and 2-Hydroxyacetophenone (0.24 ml, 2.07 mmol) were heated in acidified ethanol (20 ml) for 2 h. The solvent was removed and the product recrystallized from DMSO.

### Refinement

Hydrogen atoms were placed at calculated positions (C—H 0.95, N—H 0.88 and O—H 0.84 Å), with U(H) set to 1.2–1.5 times *U*_eq_(C,*N*,*O*).

### Crystal data

**Table:**

C_18_H_17_N_5_O_2_	*F*_000_ = 704
*M**_r_* = 335.37	*D*_x_ = 1.325 Mg m^−^^3^
Monoclinic, *P*2_1_/*n*	Mo *K*α radiation λ = 0.71073 Å
Hall symbol: -P 2yn	Cell parameters from 6889 reflections
*a* = 7.0802 (8) Å	θ = 2.3–30.5º
*b* = 9.5335 (11) Å	µ = 0.09 mm^−^^1^
*c* = 25.110 (3) Å	*T* = 100 (2) K
β = 97.295 (2)º	Irregular, colourless
*V* = 1681.2 (3) Å^3^	0.42 × 0.42 × 0.16 mm
*Z* = 4	

### Data collection

**Table:**

Bruker APEXII CCD area-detector diffractometer	4740 independent reflections
Radiation source: fine-focus sealed tube	4145 reflections with *I* > 2σ(*I*)
Monochromator: graphite	*R*_int_ = 0.014
*T* = 100(2) K	θ_max_ = 30.6º
ω scans	θ_min_ = 2.3º
Absorption correction: multi-scan(SADABS; Sheldrick, 1996)	*h* = −10→4
*T*_min_ = 0.821, *T*_max_ = 0.986	*k* = −13→12
11912 measured reflections	*l* = −34→35

### Refinement

**Table:**

Refinement on *F*^2^	Secondary atom site location: difference Fourier map
Least-squares matrix: full	Hydrogen site location: inferred from neighbouring sites
*R*[*F*^2^ > 2σ(*F*^2^)] = 0.044	H-atom parameters constrained
*wR*(*F*^2^) = 0.122	*w* = 1/[σ^2^(*F*_o_^2^) + (0.0656*P*)^2^ + 0.7125*P*] where *P* = (*F*_o_^2^ + 2*F*_c_^2^)/3
*S* = 1.03	(Δ/σ)_max_ < 0.001
4740 reflections	Δρ_max_ = 0.43 e Å^−^^3^
228 parameters	Δρ_min_ = −0.32 e Å^−^^3^
Primary atom site location: structure-invariant direct methods	Extinction correction: none

### Special details

**Table:**

Geometry. All e.s.d.'s (except the e.s.d. in the dihedral angle between two l.s. planes) are estimated using the full covariance matrix. The cell e.s.d.'s are taken into account individually in the estimation of e.s.d.'s in distances, angles and torsion angles; correlations between e.s.d.'s in cell parameters are only used when they are defined by crystal symmetry. An approximate (isotropic) treatment of cell e.s.d.'s is used for estimating e.s.d.'s involving l.s. planes.
Refinement. Refinement of *F*^2^ against ALL reflections. The weighted *R*-factor *wR* and goodness of fit *S* are based on *F*^2^, conventional *R*-factors *R* are based on *F*, with *F* set to zero for negative *F*^2^. The threshold expression of *F*^2^ > σ(*F*^2^) is used only for calculating *R*-factors(gt) *etc*. and is not relevant to the choice of reflections for refinement. *R*-factors based on *F*^2^ are statistically about twice as large as those based on *F*, and *R*- factors based on ALL data will be even larger.

### Fractional atomic coordinates and isotropic or equivalent isotropic displacement parameters (Å^2^)

**Table:**

	*x*	*y*	*z*	*U*_iso_*/*U*_eq_	
N2	0.47231 (13)	0.13948 (10)	0.11232 (3)	0.01593 (18)	
O2	0.38944 (12)	0.16315 (8)	−0.02799 (3)	0.01958 (18)	
O1	0.22435 (14)	0.47449 (9)	−0.09575 (3)	0.0246 (2)	
H1A	0.2598	0.4252	−0.0686	0.037*	
N5	0.28865 (13)	0.41919 (9)	0.00270 (3)	0.01540 (18)	
N3	0.45872 (14)	0.09630 (10)	0.05941 (4)	0.01774 (19)	
H3B	0.4878	0.0099	0.0513	0.021*	
C13	0.17966 (15)	0.64221 (11)	−0.02541 (4)	0.0159 (2)	
N4	0.35380 (14)	0.31965 (9)	0.03933 (3)	0.01631 (19)	
H4B	0.3663	0.3374	0.0740	0.020*	
C11	0.25266 (15)	0.54484 (11)	0.01820 (4)	0.0150 (2)	
C10	0.39925 (15)	0.19077 (11)	0.02031 (4)	0.0155 (2)	
C9	0.57339 (15)	0.06194 (11)	0.14689 (4)	0.0160 (2)	
H9	0.6346	−0.0193	0.1354	0.019*	
C8	0.59385 (15)	0.09842 (11)	0.20307 (4)	0.0153 (2)	
N1	0.69458 (15)	0.09189 (10)	0.29159 (4)	0.0203 (2)	
H1B	0.7590	0.0676	0.3225	0.024*	
C12	0.28004 (18)	0.59167 (13)	0.07571 (5)	0.0224 (2)	
H12A	0.1593	0.5833	0.0907	0.034*	
H12B	0.3223	0.6896	0.0777	0.034*	
H12C	0.3764	0.5326	0.0963	0.034*	
C14	0.16631 (16)	0.60243 (11)	−0.07989 (4)	0.0180 (2)	
C15	0.09241 (18)	0.69504 (13)	−0.12025 (5)	0.0237 (2)	
H15	0.0813	0.6664	−0.1568	0.028*	
C7	0.71448 (17)	0.03294 (12)	0.24298 (4)	0.0192 (2)	
H7	0.7986	−0.0418	0.2375	0.023*	
C5	0.49227 (15)	0.20425 (11)	0.22913 (4)	0.0147 (2)	
C18	0.12097 (18)	0.77799 (12)	−0.01393 (5)	0.0240 (2)	
H18	0.1294	0.8076	0.0224	0.029*	
C3	0.48431 (17)	0.27906 (12)	0.32274 (5)	0.0208 (2)	
H3	0.5303	0.2721	0.3599	0.025*	
C1	0.27606 (18)	0.38481 (13)	0.24917 (5)	0.0255 (3)	
H1	0.1791	0.4509	0.2376	0.031*	
C4	0.55764 (16)	0.19532 (11)	0.28456 (4)	0.0164 (2)	
C2	0.34160 (18)	0.37266 (13)	0.30407 (5)	0.0247 (2)	
H2	0.2868	0.4299	0.3290	0.030*	
C17	0.05073 (19)	0.87046 (13)	−0.05440 (6)	0.0283 (3)	
H17	0.0134	0.9625	−0.0457	0.034*	
C6	0.35025 (16)	0.30202 (12)	0.21144 (5)	0.0200 (2)	
H6	0.3056	0.3114	0.1743	0.024*	
C16	0.03524 (18)	0.82812 (13)	−0.10749 (5)	0.0264 (3)	
H16	−0.0148	0.8907	−0.1352	0.032*	

### Atomic displacement parameters (Å^2^)

**Table:**

	*U*^11^	*U*^22^	*U*^33^	*U*^12^	*U*^13^	*U*^23^
N2	0.0201 (4)	0.0163 (4)	0.0114 (4)	0.0015 (3)	0.0021 (3)	−0.0005 (3)
O2	0.0286 (4)	0.0170 (4)	0.0127 (3)	0.0047 (3)	0.0009 (3)	−0.0006 (3)
O1	0.0389 (5)	0.0196 (4)	0.0140 (4)	0.0084 (4)	−0.0020 (3)	−0.0002 (3)
N5	0.0172 (4)	0.0140 (4)	0.0148 (4)	0.0021 (3)	0.0012 (3)	0.0020 (3)
N3	0.0264 (5)	0.0145 (4)	0.0121 (4)	0.0054 (3)	0.0016 (3)	−0.0001 (3)
C13	0.0141 (4)	0.0142 (4)	0.0192 (5)	0.0006 (4)	0.0015 (4)	0.0011 (4)
N4	0.0228 (5)	0.0140 (4)	0.0118 (4)	0.0046 (3)	0.0008 (3)	0.0009 (3)
C11	0.0144 (4)	0.0146 (4)	0.0162 (4)	0.0004 (4)	0.0027 (3)	−0.0004 (4)
C10	0.0175 (5)	0.0144 (4)	0.0143 (4)	0.0017 (4)	0.0013 (4)	0.0002 (3)
C9	0.0180 (5)	0.0149 (4)	0.0153 (4)	0.0021 (4)	0.0033 (4)	0.0004 (4)
C8	0.0172 (5)	0.0148 (4)	0.0139 (4)	0.0013 (4)	0.0024 (4)	0.0012 (3)
N1	0.0262 (5)	0.0209 (5)	0.0128 (4)	0.0031 (4)	−0.0008 (3)	0.0018 (3)
C12	0.0300 (6)	0.0201 (5)	0.0173 (5)	0.0019 (4)	0.0036 (4)	−0.0036 (4)
C14	0.0173 (5)	0.0162 (5)	0.0197 (5)	0.0010 (4)	−0.0007 (4)	0.0018 (4)
C15	0.0235 (6)	0.0242 (6)	0.0221 (5)	0.0013 (4)	−0.0028 (4)	0.0061 (4)
C7	0.0232 (5)	0.0187 (5)	0.0157 (5)	0.0044 (4)	0.0016 (4)	0.0015 (4)
C5	0.0161 (5)	0.0138 (4)	0.0143 (4)	−0.0022 (4)	0.0027 (3)	−0.0007 (3)
C18	0.0265 (6)	0.0172 (5)	0.0281 (6)	0.0045 (4)	0.0033 (5)	−0.0003 (4)
C3	0.0244 (6)	0.0212 (5)	0.0176 (5)	−0.0058 (4)	0.0052 (4)	−0.0048 (4)
C1	0.0210 (6)	0.0239 (6)	0.0310 (6)	0.0058 (4)	0.0013 (5)	−0.0083 (5)
C4	0.0188 (5)	0.0151 (5)	0.0156 (5)	−0.0030 (4)	0.0030 (4)	−0.0003 (4)
C2	0.0240 (6)	0.0244 (6)	0.0269 (6)	−0.0022 (5)	0.0076 (5)	−0.0108 (5)
C17	0.0284 (6)	0.0161 (5)	0.0402 (7)	0.0068 (4)	0.0034 (5)	0.0046 (5)
C6	0.0189 (5)	0.0199 (5)	0.0208 (5)	0.0031 (4)	0.0002 (4)	−0.0029 (4)
C16	0.0219 (6)	0.0218 (6)	0.0343 (6)	0.0021 (4)	−0.0013 (5)	0.0116 (5)

### Geometric parameters (Å, °)

**Table:**

N2—C9	1.2862 (14)	C12—H12A	0.9800
N2—N3	1.3824 (12)	C12—H12B	0.9800
O2—C10	1.2344 (13)	C12—H12C	0.9800
O1—C14	1.3626 (13)	C14—C15	1.3949 (15)
O1—H1A	0.8400	C15—C16	1.3816 (18)
N5—C11	1.2948 (13)	C15—H15	0.9500
N5—N4	1.3608 (12)	C7—H7	0.9500
N3—C10	1.3591 (13)	C5—C6	1.4013 (15)
N3—H3B	0.8800	C5—C4	1.4125 (14)
C13—C18	1.4007 (15)	C18—C17	1.3887 (17)
C13—C14	1.4111 (15)	C18—H18	0.9500
C13—C11	1.4779 (14)	C3—C2	1.3844 (18)
N4—C10	1.3712 (13)	C3—C4	1.3973 (15)
N4—H4B	0.8800	C3—H3	0.9500
C11—C12	1.5003 (15)	C1—C6	1.3866 (16)
C9—C8	1.4424 (14)	C1—C2	1.4024 (18)
C9—H9	0.9500	C1—H1	0.9500
C8—C7	1.3807 (14)	C2—H2	0.9500
C8—C5	1.4431 (14)	C17—C16	1.384 (2)
N1—C7	1.3672 (14)	C17—H17	0.9500
N1—C4	1.3784 (14)	C6—H6	0.9500
N1—H1B	0.8800	C16—H16	0.9500
			
C9—N2—N3	116.25 (9)	C15—C14—C13	120.46 (10)
C14—O1—H1A	109.5	C16—C15—C14	120.45 (11)
C11—N5—N4	120.31 (9)	C16—C15—H15	119.8
C10—N3—N2	118.31 (9)	C14—C15—H15	119.8
C10—N3—H3B	120.8	N1—C7—C8	109.75 (10)
N2—N3—H3B	120.8	N1—C7—H7	125.1
C18—C13—C14	117.56 (10)	C8—C7—H7	125.1
C18—C13—C11	120.87 (10)	C6—C5—C4	118.98 (10)
C14—C13—C11	121.57 (9)	C6—C5—C8	134.45 (10)
N5—N4—C10	117.64 (9)	C4—C5—C8	106.55 (9)
N5—N4—H4B	121.2	C17—C18—C13	121.61 (12)
C10—N4—H4B	121.2	C17—C18—H18	119.2
N5—C11—C13	114.95 (9)	C13—C18—H18	119.2
N5—C11—C12	123.94 (10)	C2—C3—C4	117.05 (10)
C13—C11—C12	121.10 (9)	C2—C3—H3	121.5
O2—C10—N3	122.82 (10)	C4—C3—H3	121.5
O2—C10—N4	123.15 (9)	C6—C1—C2	121.18 (11)
N3—C10—N4	114.02 (9)	C6—C1—H1	119.4
N2—C9—C8	119.98 (10)	C2—C1—H1	119.4
N2—C9—H9	120.0	N1—C4—C3	129.59 (10)
C8—C9—H9	120.0	N1—C4—C5	107.85 (9)
C7—C8—C9	125.24 (10)	C3—C4—C5	122.54 (10)
C7—C8—C5	106.56 (9)	C3—C2—C1	121.48 (11)
C9—C8—C5	128.18 (9)	C3—C2—H2	119.3
C7—N1—C4	109.28 (9)	C1—C2—H2	119.3
C7—N1—H1B	125.4	C16—C17—C18	119.80 (11)
C4—N1—H1B	125.4	C16—C17—H17	120.1
C11—C12—H12A	109.5	C18—C17—H17	120.1
C11—C12—H12B	109.5	C1—C6—C5	118.75 (11)
H12A—C12—H12B	109.5	C1—C6—H6	120.6
C11—C12—H12C	109.5	C5—C6—H6	120.6
H12A—C12—H12C	109.5	C15—C16—C17	120.09 (11)
H12B—C12—H12C	109.5	C15—C16—H16	120.0
O1—C14—C15	116.97 (10)	C17—C16—H16	120.0
O1—C14—C13	122.57 (9)		
			
C9—N2—N3—C10	−162.57 (10)	C5—C8—C7—N1	−0.35 (13)
C11—N5—N4—C10	−176.52 (10)	C7—C8—C5—C6	−178.47 (12)
N4—N5—C11—C13	−179.00 (9)	C9—C8—C5—C6	−0.3 (2)
N4—N5—C11—C12	0.64 (16)	C7—C8—C5—C4	−0.58 (12)
C18—C13—C11—N5	174.99 (10)	C9—C8—C5—C4	177.62 (10)
C14—C13—C11—N5	−5.00 (15)	C14—C13—C18—C17	0.46 (18)
C18—C13—C11—C12	−4.66 (16)	C11—C13—C18—C17	−179.52 (11)
C14—C13—C11—C12	175.35 (10)	C7—N1—C4—C3	177.00 (11)
N2—N3—C10—O2	177.29 (10)	C7—N1—C4—C5	−1.54 (13)
N2—N3—C10—N4	−1.86 (15)	C2—C3—C4—N1	−178.03 (11)
N5—N4—C10—O2	2.37 (16)	C2—C3—C4—C5	0.33 (17)
N5—N4—C10—N3	−178.48 (9)	C6—C5—C4—N1	179.57 (10)
N3—N2—C9—C8	−179.42 (9)	C8—C5—C4—N1	1.29 (12)
N2—C9—C8—C7	−172.45 (11)	C6—C5—C4—C3	0.90 (16)
N2—C9—C8—C5	9.66 (17)	C8—C5—C4—C3	−177.38 (10)
C18—C13—C14—O1	178.09 (11)	C4—C3—C2—C1	−1.19 (18)
C11—C13—C14—O1	−1.93 (17)	C6—C1—C2—C3	0.8 (2)
C18—C13—C14—C15	−1.62 (16)	C13—C18—C17—C16	0.9 (2)
C11—C13—C14—C15	178.37 (10)	C2—C1—C6—C5	0.44 (18)
O1—C14—C15—C16	−178.26 (11)	C4—C5—C6—C1	−1.26 (16)
C13—C14—C15—C16	1.46 (18)	C8—C5—C6—C1	176.42 (12)
C4—N1—C7—C8	1.19 (13)	C14—C15—C16—C17	−0.10 (19)
C9—C8—C7—N1	−178.62 (10)	C18—C17—C16—C15	−1.1 (2)

### Hydrogen-bond geometry (Å, °)

**Table:**

*D*—H···*A*	*D*—H	H···*A*	*D*···*A*	*D*—H···*A*
O1—H1A···N5	0.84	1.78	2.5121 (11)	145
N3—H3B···O2^i^	0.88	1.99	2.8481 (13)	166
N1—H1B···O1^ii^	0.88	2.14	2.8803 (13)	142

Symmetry codes: (i) −*x*+1, −*y*, −*z*; (ii) *x*+1/2, −*y*+1/2, *z*+1/2.
